# Treatment of chronic pain associated with nocturnal bruxism with botulinum toxin. A prospective and randomized clinical study

**DOI:** 10.4317/jced.53084

**Published:** 2017-01-01

**Authors:** Hessa Al-Wayli

**Affiliations:** 1 Consultant, Dept. of Oral Medicine, Head of preventive dental department, Dental Administration, Riyadh Health, Riyadh, Saudi Arabia

## Abstract

**Background:**

To evaluate the role of botulinum toxin type A (BTX-A) in the treatment of pain associated with nocturnal bruxism.

**Material and Methods:**

Fifty subjects reporting nocturnal bruxism were recruited for a randomized clinical trial. Twenty five bruxers were injected with botulinum toxin in both masseters, and twenty five were treated with traditional methods of treating bruxism. Patients were evaluated at 3rd week, 2nd and 6th month and one year after injection and then used to calculate bruxism events. Bruxism symptoms were investigated using questionnaires.

**Results:**

Mean pain score due to Bruxism events in the masseter muscle decreased significantly in the botulinum toxin injection group A (*P* =0.000, highly significant). However, in the conventional treatment group, mean pain score does not show improvement with time (*p*>0.05).

**Conclusions:**

Our results suggest that botulinum toxin injection reduced the mean pain score and number of bruxism events, most likely by decreasing the muscle activity of masseter rather than affecting the central nervous system.

** Key words:**Temporomandibular pain, nocturnal bruxism, botulinum toxin.

## Introduction

Bruxism, which includes clenching or grinding of the teeth, or both, affects from 50% to 95% of the adult population (1-3). Bruxism is caused by the activation of reflex chewing activity. Various forms of bruxism have been described (4,5). The etiology of this disorder is uncertain. Some experts believe that it is related to anxiety and stress. Other explanations include asymmetry of teeth, and digestive and sleep disturbances. Bruxism can affect the muscles solely or can act as a parafunction that is an initiating and/or perpetuating factor in more involved forms of temporomandibular disorders (TMD)involving joint damage. The treatment of bruxism includes behavioural therapy, dental appliances, and medications (6-8). Many characteristics of bruxism mimic those of dystonia, including similar epidemiology, pain, and exacerbation by external factors such as fatigue, stress, and emotional stress. Several experts have suggested that bruxism may itself be a form of focal dystonia (9). If bruxism is a type of dystonia, it is possible that the success of the most common treatment of bruxism, with intraoral appliances or occlusal adjustments, may simply be a ‘‘sensory trick’’ that relieves dystonicsymptoms. Regardless of the etiology of bruxism, successful use of Botulinum Toxin for bruxism has been described (10-13).

According to the American Sleep Disorders Association, the diagnosis of nocturnal bruxism is based on the reports of tooth grinding or clenching and one of the following signs: abnormal tooth wear, sounds associated with bruxism, and jaw muscle discomfort. Bruxism can also produce an increase in dental wear and temporomandibular dysfunction. Delaying treatment, in some cases, may result in luxation and degenerative arthritis of the temporomandibular joint (14). In order to prevent these complications, the early diagnosis, as well as the appropriate treatment, is very important. The current therapies for this dysfunction are not totally effective. In lieu of this, the aim of the present study is to evaluate the role of botulinum toxin type A (BTX-A) in the treatment of pain associated with nocturnal bruxism when compared to traditional methods of treatment.

## Material and Methods

A prospective, randomized, controlled, parallel group design study was conducted with the approbation of the Department of Dentistry, Alyamamah Hospital, Riyadh, Saudi Arabia over the period of a year from December, 2010 to December, 2011. The study protocol was reviewed and approved by the Institutional Ethical Committee, and the Helsinki guidelines were followed. The procedures were explained to the patients verbally and in writing, and informed consent was taken before enrolment. Those who were not ready or fail to report according to the set criteria were excluded from the study. The patients were randomly allocated to two group’s i.e. experimental group I using BTX-A administration group and control group II that used traditional methods of treatment of Bruxism.

All patients (females) with bruxism associated with chronic pain in masseter muscles bilaterally participated in this study. The mean ages were 45.5± 10.8 years.

According to the diagnostic grading system of bruxism [Lobbezoo *et al.* (14)] ( Table 1), all subjects underwent an assessment including a bruxism questionnaire ( Table 2) (i.e., oral history taking with specific focus on bruxism habits) plus a clinical examination to evaluate bruxism signs and symptoms. All patients were diagnosed as ‘probable’ sleep bruxism based on self-report plus the inspection part of a clinical examination.

**Table 1:**
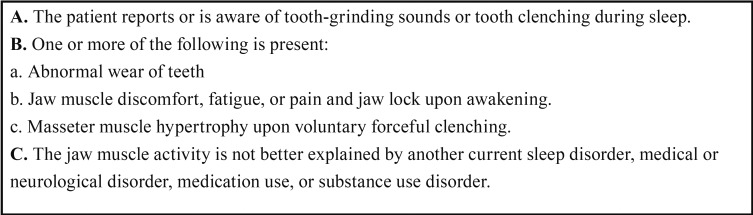
Nocturnal Bruxism Criteria according to International Classification of Sleep Disorders, Second Edition (ICSD-2).

**Table 2:**
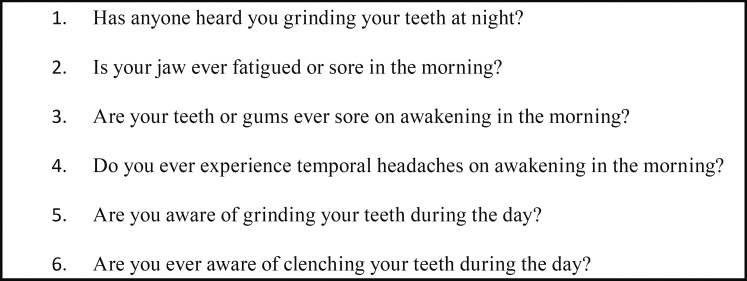
Questionnaire for detecting Bruxers.

The inclusion criteria for this study were as follows:

1. Moderate to severe pain in relation to the masseter muscles and TMJ area related to bruxism during clinical examination.

2. Aged 20-60 patients.

3. Tooth-grinding sounds corroborated by family members or caregivers.

4. Cases where bruxism resulted in occlusal surface attrition of posterior teeth; and

Exclusion criteria were pain in the orofacial region, insomnia, known botulinum toxin allergy, pregnancy, neuromuscular disease, bleeding disorders, antibiotic therapy, pulmonary disease that produced coughing during sleep, or infectious skin lesion at the site of the injection.

The treatment plan was explained to all the study participants, and their consent was obtained. A total of 50 patients included in the study were randomly and equally divided into two groups.

**Figure 1:**
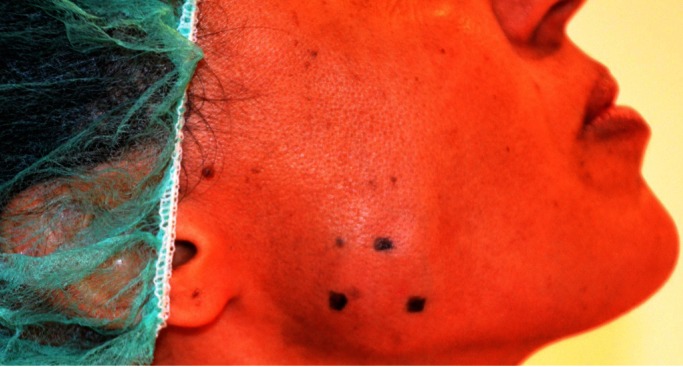
Points of application of BOTOX in the masseter muscle.

Group I (25) patients underwent treatment with 20 units of BOTOX, (Allergan Inc.) per side was injected at three points into masseter muscle bilaterally (Fig. 1).

Group II (25) patients were treated with conventional method of treating bruxism with the use of behavioural strategies which includes reassurance and detailed explanation of the nature of the disease, occlusal splints, and pharmacologic measures.

All patients had initially had a two months course of conservative treatment, which included reassurance and explanation of the nature of the problem, supplemented in a written format; advice on rest, soft diet, and how to avoid clenching; advice on the regular use of systemic non-steroidal anti-inflammatory drugs such as diclofenac sodium (50mg bid), and the use of a lower soft bite-raising appliance for a minimum of 8 weeks. Those who did not respond to conservative measures on subsequent review were given the following options: persevere with conservative measures; or have botulinum injections into the masseter muscle bilaterally.

Outcomes for injections were assessed using 10 cm visual analogue pain scores (VAS) at 0, 3rd week, 2 months 6 months and 1 year. Once patients had consented to treatment with botulinumtoxin, they indicated their average pain for the previous weekon a VAS sliding ruler for both sides of the face. They then had up to 20 units of BOTOX injected into masseter muscles bilaterally. All patients were reviewed 3 weeks after injection and then again recorded their average pain levels for the previous week using the same VAS ruler. If free from pain they were discharged to primary care and told to return to clinic if unprecedented events occurs. We considered our true primaryend point to be a reduction in pain of 90% or more. The outcome was the proportion of patients whose pain had reduced at one year review, which was measured by taking the difference in the four scores (VAS) as a percentage of the score before injection.

All patients were evaluated at 3 weeks, 2 months, 6 months, and 1 year.

-Statistical analysis

Evaluation of the data was done using the statistical package for the social science (SPSS 17.0, Illinois, Chicago) using student unpaired t- test and Wilcoxon sign rank test. *P*< 0.05 consider statistically significant.

## Results

The present study was done in order to estimate the improvement in pain score related to the bruxism patients especially in the masseter muscles, when treated with BOTOX and traditional methods. The results were evaluated using student unpaired t test and Wilcoxon sign rank test.

The mean pain score pre-operatively in group I was 7.1 ± 0.72 and in group B was 7.5 ± 0.66. There was no significant difference in mean pain score pre-operatively in group I and group II. Mean pain score post-operatively at 3 weeks in group I was 4.6 ± 0.58 and in group II was 5.4 ± 0.58.There was highly significant difference in mean pain score post-operatively at 3 weeks in group I and group II (*p*= 0.000). The mean pain score at 2nd month post-operatively in group I was 2.5 ± 0.59 and in group II was 4.3 ± 0.48. There was highly significant difference in mean pain score at post-operative 2nd month in group I and group II. Mean pain score at 6th month and 1 year post-operatively in group I was 0.2 ± 0.51 and in group II was 2.1 ± 0.74. There was highly significant difference in mean pain score post-operatively at 6th month and 1 year in group I and group II ( Table 3, Table 4).

**Table 3:**
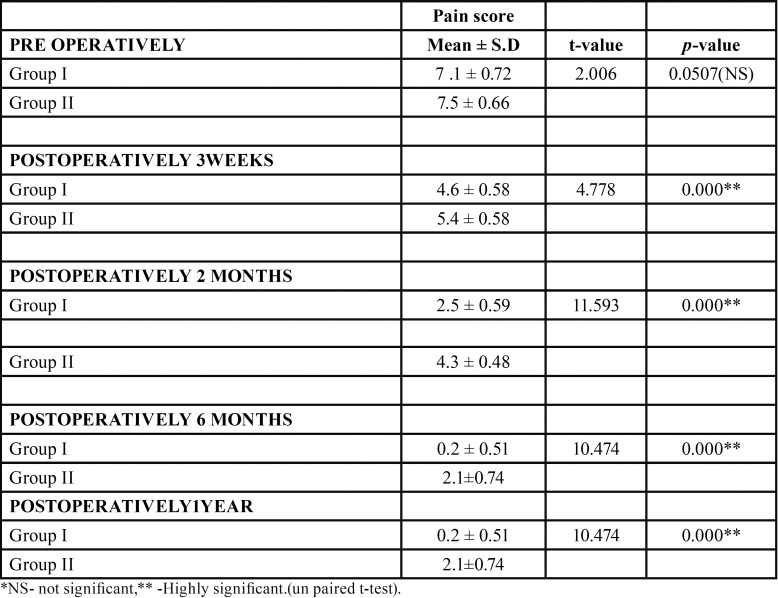
Pain scores using unpaired t test at 3 weeks, 2 months, 6 months, and 1 year post-operatively.

**Table 4:**
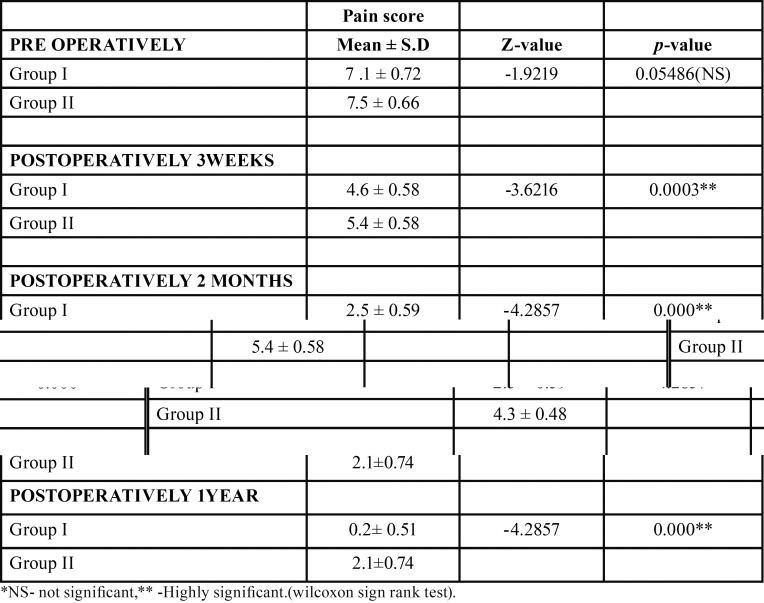
Pain scores using Wilcoxon sign rank test at 3 weeks, 2 months, 6 months, and 1 year post-operatively.

## Discussion

Botulinum Toxin is the most powerful known neurotoxin, produced by anaerobic gram-positive bacteria, Clostridium botulinum.This was described for the first time in 1817 by JC Kerner, but it was not until 1895 that the cause of botulism was high lighted by Van Ermengem, and the type A toxin was finally isolated by Sommer in 1920. Only after the Second World War did work on the different forms of toxin really begin, and their practical application into human pathology only began at the end of the 1970’s with the work of Scott in the treatment of strabismus (15,16). Seven toxin serotypes (A to G) are currently known, only three of them (A, B and E) seem to be toxic to mankind. The toxin acts by causing a sort of chemical denervation by blocking neurotransmitter release at the synaptic cleft of the acetylcholine fibers of the motor nerves and of the autonomic nervous system (17). It can thus be used as a local acting anti-acetylcholine agent. On contrary basis, two other anti-acetylcholine drugs such as atropine or probanthine, botulinum toxin has few side effects, and these are always restricted to the area of injection (allergic reaction ormuscular weakness) (18).

Due to different taxonomies and diagnostic aspects, thre is some difficulty in determining an acceptable standardization of diagnosis for bruxism (19). The American Academy of Sleep Medicine defines bruxism as astereotyped oral and motor sleep disorder characterized by the teeth grinding and tightening, while the American Academy for Orofacial Pain extends the definition to the same movements that occur in the waking state. Intramuscular applications of BOTOX arean effective treatment for a variety of movement afflictions (20). They inhibit the exocytotic release of acetylcholine in the motor nerve terminals leading to reduced muscle contraction. This property makes it useful both clinically and therapeutically for a series of conditions where there is an excess of muscle contraction (20). Recent advances show that bruxism is caused by high levels of motor activity in the centrally situated mandible musculature, indicating that the reduction in muscle activity induced by the use of BOTOX could be beneficial in these cases (21).

The masseters were injected because they are the chief muscles involved in the repetitive grinding movements seen with bruxism (22). The other muscles of mastication (ie, temporalis, medial and lateral pterygoid, digastric, and geniohyoid) were not treated so that chewing and swallowing could occur. This is a different approach from previous reports of successful bruxism treatment in which both the masseter and temporalis muscles were injected (23,24). Masseter injection alone resulted in a similar therapeutic response and, we believe, was easier to perform without the need for general anesthesia.

This suggests that the application of botulinum toxin type A reduces the number of bruxism events, probably due to diminishing of peripheral muscle activity, without presenting an action on the central nervous system.

It is quite interesting that how a single dose of BTX-A injection in the masseter muscle totally abolishes severe bruxing behaviour, as depicted in our study, is conniving. Nonetheless, it has been hypothesized that jaw muscle paralysis induced by BTX A may disrupt the feed back loop from the trigeminal motor nucleus and inhibit the central bruxism generator. Alternatively, it also may deactivate periodontal mechano receptors during mastication, which have been thought to have a facilitatory effect on jaw closure motor neurons (25).

Guarda-Nardini *et al.* (26) compared the efficacy of botulinum toxin with the saline solution in the reduction of pain in 20 patients with bruxism and myofascial pain in the masticatory muscles. The pain levels at rest and in mastication were evaluated through the visual analogue scale (VAS) in the interval of 0-10, before and after the application with botulinum toxin. The authors injected 30UI of botulinum toxin type A (BOTOX®, Allergan) at three points in the masseter muscles and 20UI at two points in the anterior temporalis muscles of 10 patients with myofascial pain associated with bruxism, and they used saline solution on the remaining selected patients. They observed that the degree of pain reduction in mastication, over the course of six months of follow-up, was significantly greater in the botulinum toxin group than in the placebo group. However, in our study we have injected 20 units of BOTOX in the masseter muscle bilaterally. Moreover, the follow-up period was at 3rd week, 2 months, 6 months and 1 year with profound improvement in group I in pain as compared to the group II (*p*<0.05).

Lee *et al.* (27) compared the efficacy of botulinum toxin with the placebo in reducing the frequency of bruxism events after the application (4, 8, and 12 weeks after the application) in 12 patients with bruxism. Those authors injected 80UI of botulinum toxin A (Dysport®)at three points in both masseter muscles in six patients, comparing them with the other six patients who received applications of saline solution. They observed that the patients treated with botulinum toxin showed a significant reduction of the masseter muscle electromyographic activity and clinical improvement of bruxism, while the temporalis muscle activity was not altered. Through electromyography (EMG) it was detected that the bruxism was significantly less frequent in the group that received botulinum toxin A than in the group that received placebo. Their results suggest that botulinum toxin reduces the number of bruxism events by reducing muscle activity, concluding that it is an effective treatment for nocturnal bruxism. Similarly, all of our patients in group I were treated effectively for bruxism with low dose of BOTOX (20 Units) per side.

The above mentioned studies were the only randomized clinical trial till now available in the literature, however, our study is the only study on the Saudi female population as it was tertiary referral centre for females only. Further, larger sample sized, meta-analysis studies are required to validate the findings of our study for the treatment of Bruxism associated with myofascial and TMD pain.

Several complications after botulinum toxin injection into the masticatory muscles have been reported, including mastication difficulties, muscle pain, speech disturbance, and unnatural facial appearance. But these complications are reported to be transient, usually lasting from 1 to 4 weeks after injection (28). Immunologic responses such as allergic skin reactions or formation of antibodies can occur in a small percentage of subjects (29). However, we did not observe any of these problems in our sample.

20 UI per side Botulinum toxin injection in the masseter muscles is an effective and safe means of intervention in cases of moderate to severe chronic myofascial and TMJ pain associated with bruxism. The patient should be evaluated 15 days after the application and return for control after three or four months after the application for a new evaluation and another application, if needed. In this way, the treatment of bruxism with botulinum toxin type A can present itself as a possible treatment for bruxism patients. More studies are needed that follow the quality criteria to reach a definitive conclusion on safety and efficacy.

## References

[1] Tan EK, Jankovic J, Ondo W (2000). Bruxism in Huntington's disease. Mov Disord.

[2] Kim HS, Yun PY, Kim YK (2016). A clinical evaluation of botulinum toxin-A injections in the temporomandibular disorder treatment. Maxillofac Plast Reconstr Surg.

[3] Tinastepe N, Küçük BB, Oral K (2015). Botulinum toxin for the treatment of bruxism. Cranio.

[4] Tinastepe N, Küçük BB, Oral K (2014). Botulinum toxin for the treatment of bruxism. Cranio.

[5] Sevim S, Kaleağası H, Fidancı H (2015). Sleep bruxism possibly triggered by multiple sclerosis attacks and treated successfully with botulinum toxin: Report of three cases. Mult Scler Relat Disord.

[6] Azam A, Manchanda S, Thotapalli S, Kotha SB (2015). Botox Therapy in Dentistry: A Review. J Int Oral Health.

[7] Malcmacher L (2015). Bruxism--Are You Helping or Hurting Your Patients?. J N J Dent Assoc.

[8] Manfredini D, Ahlberg J, Winocur E, Lobbezoo F (2015). Management of sleep bruxism in adults: a qualitative systematic literature review. J Oral Rehabil.

[9] Watts MW, Tan EK, Jankovic J (1999). Bruxism and cranial-cervical dystonia: is there a relationship?. Cranio.

[10] Pedemonte C, Pérez Gutiérrez H, González E, Vargas I, Lazo D (2015). Use of onabotulinumtoxinA in post-traumatic oromandibulardystonia. J Oral Maxillofac Surg.

[11] Malcmacher L (2014). Are you prescribing bruxism appliances?. Dent Today.

[12] Kesikburun S, Alaca R, Aras B, Tuğcu I, Tan AK (2014). Botulinum toxin injection for bruxism associated with brain injury: case report. J Rehabil Res Dev.

[13] Shim YJ, Lee MK, Kato T, Park HU, Heo K, Kim ST (2014). Effects of botulinum toxin on jaw motor events during sleep in sleep bruxism patients: a polysomnographicevaluation. J Clin Sleep Med.

[14] Lobbezoo F, Ahlberg J, Glaros AG, Kato T, Koyano K, Lavigne GJ (2013). Bruxism defined and graded: an international consensus. J Oral Rehabil.

[15] Walker TJ, Dayan SH (2014). Comparison and overview of currently available neurotoxins. J Clin Aesthet Dermatol.

[16] Garcia-Ruiz PJ (2013). Applications of botulinum toxin in Neurology. Med Clin (Barc).

[17] Kreyden OP, Geiges ML, Böni R, Burg G (2000). Botulinum toxin: from poison to drug. A historical review. Hautarzt.

[18] Laskin DM (2012). Botulinum toxin A in the treatment of myofascial pain and dysfunction: the case against its use. J Oral Maxillofac Surg.

[19] Manfredini D, Lobbezoo F (2010). Relationship between bruxism and temporomandibular disorders: a systematic review of literature from 1998 to 2008. Oral Surg Oral Med Oral Pathol Oral Radiol Endod.

[20] Aoki KR (2005). Review of a proposed mechanism for the antinociceptive action of botulinum toxin type A. Neurotoxicology.

[21] Long H, Liao Z, Wang Y, Liao L, Lai W (2012). Efficacy of botulinum toxins on bruxism: an evidence-based review. Int Dent J.

[22] Guaita M, Högl B (2016). Current treatments of bruxism. Curr Treat Options Neurol.

[23] Alonso-Navarro H, Jiménez-Jiménez FJ, Plaza-Nieto JF, Pilo-De la Fuente B, Navacerrada F, Arroyo-Solera M (2011). Treatment of severe bruxism with botulinum toxin type A. Rev Neurol.

[24] Finiels PJ, Batifol D (2014). The use of botulinum toxin in the treatment of the consequences of bruxism on cervical spine musculature. Toxicon.

[25] Lavigne G, Kim JS, Valiquette C, Lund JP (1987). Evidence that periodontal pressoreceptors provide positive feedback to jaw closing muscles during mastication. J Neurophysiol.

[26] Guarda-Nardini L, Manfredini D, Salamone M, Salmaso L, Tonello S, Ferronato G (2008). Efficacy of botulinum toxin in treating myofascial pain inbruxers: a controlled placebo pilot study. Cranio.

[27] Lee SJ, McCall WD Jr, Kim YK, Chung SC, Chung JW (2010). Effect of botulinum toxin injection on nocturnal bruxism: a randomized controlled trial. Am J Phys Med Rehabil.

[28] Klein FH, Brenner FM, Sato MS, Robert FM, Helmer KA (2014). Lower facial remodeling with botulinum toxin type A for the treatment of masseter hypertrophy. An Bras Dermatol.

[29] Borodic G (2007). Botulinum toxin, immunologic considerations with long-term repeated use, with emphasis on cosmetic applications. Facial Plast Surg Clin North Am.

